# Case Report: Fabrication of a plumper-type music splint for saxophone performance in a patient with a full-arch implant-supported prosthesis

**DOI:** 10.3389/fdmed.2026.1767272

**Published:** 2026-03-11

**Authors:** Mariko Hattori, Yuichi Yamatani, Yuka Sumita, Noriyuki Wakabayashi

**Affiliations:** 1 Department of Advanced Prosthodontics, Division of Oral Health Sciences, Graduate School of Medical and Dental Sciences, Institute of Science Tokyo, Tokyo, Japan; 2 Dental Laboratory, Institute of Science Tokyo Hospital, Tokyo, Japan; 3 Department of Partial and Complete Denture, School of Life Dentistry at Tokyo, The Nippon Dental University, Tokyo, Japan

**Keywords:** full-arch implant, functional assessment, maxillofacial prosthesis, music splint, oral motor function, patient-reported outcomes, saxophone performance, wind instrument playing

## Abstract

**Introduction:**

The orofacial region, particularly the lips, tongue, and teeth, plays a critical role in wind instrument performance. Wind instrument players may experience lip pain, trauma, or discomfort during performance. In patients rehabilitated with fixed implant-supported full-arch prostheses, reduced soft tissue support and intraoral volume may compromise embouchure stability and endurance.

**Case description:**

A 55-year-old male amateur saxophone player reported early fatigue and difficulty maintaining embouchure stability after receiving a fixed implant-supported full-arch prosthesis. Clinical examination revealed a space between the prosthetic superstructure, residual alveolar ridge, and buccal mucosa. To compensate for the reduced intraoral tissue volume, a plumper-type music splint worn exclusively during instrument playing was planned. The appliance was initially fabricated as a provisional music splint, digitized using a model scanner, and finalized through computer-controlled milling of acrylic resin. Subjective evaluation of playing comfort was conducted using an exploratory 10-point scale, and maximum sustained note duration was measured.

**Results:**

One month after delivery, the splint showed good fit and resulted in marked improvement in playing comfort and reduction of fatigue. Performance-related functions that had been severely compromised without the splint, including buccal stability and low-register tone production, improved to clinically meaningful levels. After one year of follow-up, further improvements were observed, and the patient reported increased ease and confidence during performance, accompanied by prolonged maximum sustained note duration.

**Conclusion:**

The plumper-type music splint effectively compensated for insufficient intraoral tissue volume caused by the existing implant-supported prosthesis and enhanced embouchure stability and playing comfort. This removable, low-risk appliance may represent a useful adjunct for wind instrument players who experience performance-related functional impairment following fixed full-arch implant rehabilitation.

## 1 Introduction

The orofacial region, particularly the lips, tongue, and teeth, plays an important role in wind instrument performance (1, 2). Wind instrument players may experience pain, trauma, or discomfort of the lips, occasionally leading to ulceration (3, 4). Appliances designed to protect the lips during playing have long been described in the literature. Porter introduced a lip shield for woodwind players (5), and similar devices have been referred to as embouchure aids, intended to reduce lip irritation during performance (6). The term music splint is a collective designation for removable intraoral performance-assist devices used during wind instrument playing (7). Previous studies have demonstrated that music splints can influence both the produced sound and the player's subjective evaluation of performance (4).

In addition to performance aids, prosthetic dental treatment is sometimes required for wind musicians who have lost teeth in order to continue their musical career. Removable prostheses have been reported in the literature for such patients (8), and implant-supported prostheses have also been applied in cases of partial tooth loss with consideration of musical performance (9). In recent years, however, fixed prosthetic rehabilitation using dental implants has become more common than removable prostheses, even in full-arch cases. Although fixed implant-supported prostheses offer many benefits, the lack of a denture base often results in insufficient restoration of alveolar bone and soft tissue volume, which can affect speech (10), facial contour, and potentially wind instrument performance.

A plumper is a removable maxillofacial prosthesis designed to provide additional bulk to the soft tissues. It is commonly used in patients with maxillofacial defects who require increased lip support following tumor resection and reconstructive surgery (11) or in cases of congenital malformation (12). The concept of a plumper was adapted in the present case, in which a patient experienced difficulty playing the saxophone after full-mouth rehabilitation with an implant-supported prosthesis. The plumper used in this patient was a plumper-type music splint, designed specifically for musical performance and worn only during instrument playing. The condition was successfully managed with this appliance, which is described in this report.

## 2 Case description

A 55-year-old male amateur saxophone player presented with complaints of early fatigue during instrument performance. He reported that this difficulty had developed after receiving a fixed implant-supported full-arch prosthesis in the maxilla at another clinic. The patient regularly played the alto saxophone as a member of an amateur orchestra. His embouchure was of the single-lip type, and his principal playing style also relied on single-lip technique.

### 2.1 Diagnostic assessment

**Figure 1:**
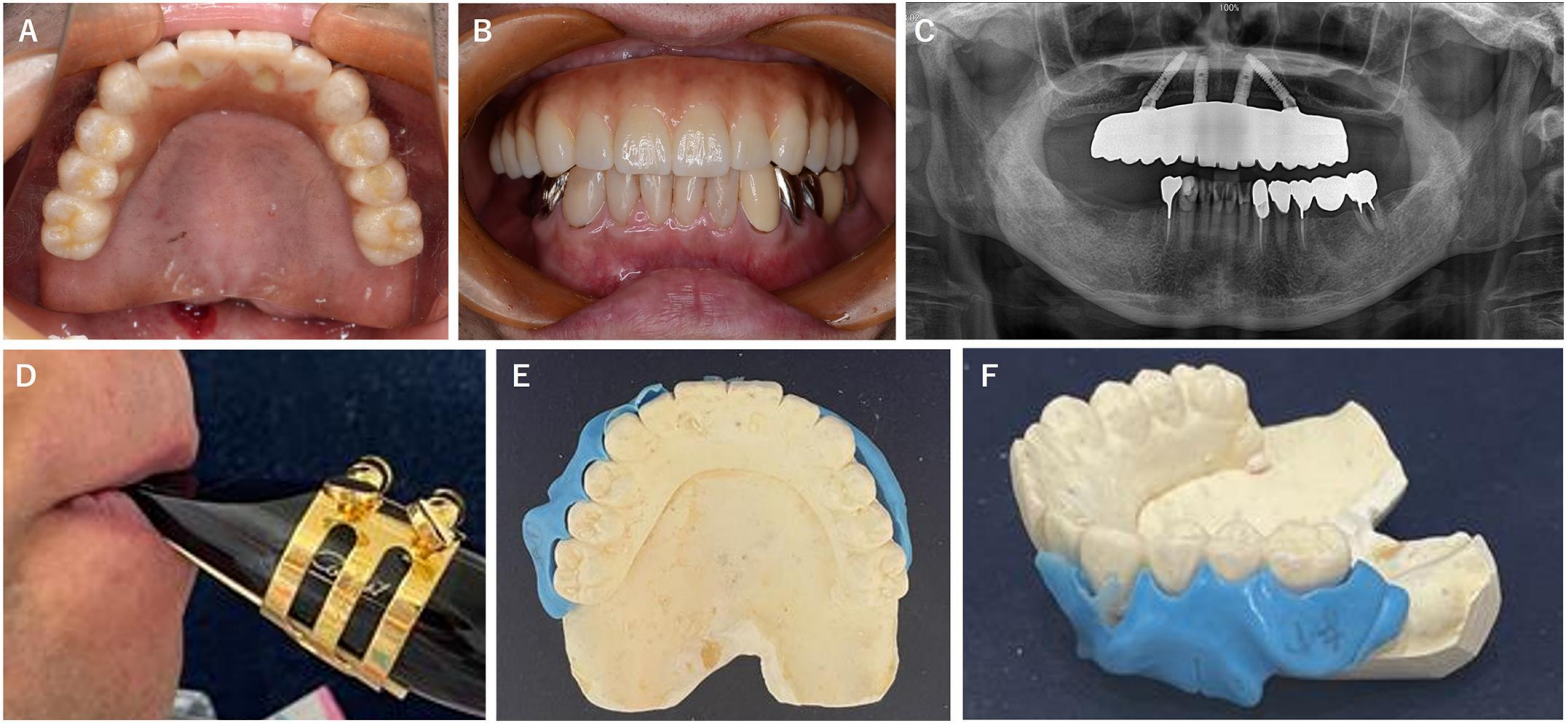
The patient's condition at the first visit. **(A)** Intraoral occlusal view. **(B)** Intraoral frontal view. **(C)** Panoramic x-ray of the upper and lower jaws. **(D)** The embouchure while the patient was playing the clarinet. **(E)** Lateral dental impression on the maxillary model, occlusal view. **(F)** Lateral dental impression on the maxillary model, lateral view.

Intraoral examination revealed a completely edentulous maxilla restored with a fixed implant-supported prosthesis (Figures 1A–C). A space was observed between the prosthetic superstructure, the residual alveolar ridge, and the buccal mucosa. To evaluate the embouchure condition during performance, a functional impression was made using silicone impression material while the patient played (13). This procedure revealed a pronounced lateral embouchure gap, particularly on the right side (Figures 1D–F).

Based on these findings, a treatment plan was formulated to fabricate a plumper-type music splint designed to fill the existing space and provide improved support during saxophone performance. While reconstruction of the prosthetic superstructure was considered, it was deemed unnecessary, as the difficulty occurred only during saxophone playing and the existing prosthesis functioned adequately in daily activities; furthermore, remaking the superstructure would have imposed a significant financial burden.

### 2.2 Therapeutic intervention

**Figure 2:**
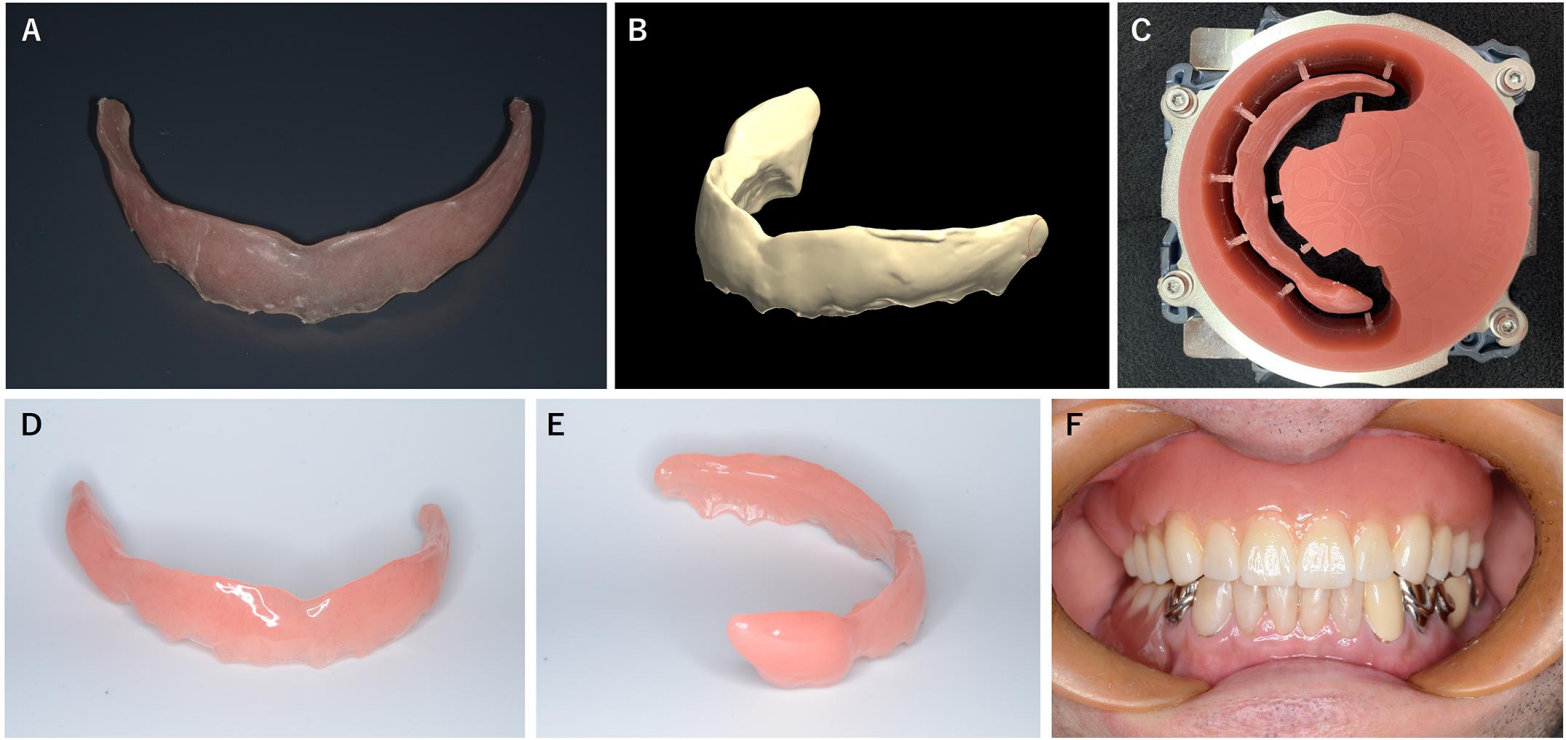
The fabrication of the definitive music splint using the data from the provisional splint. **(A)** Provisional music splint. **(B)** Digitized surface data of the provisional splint. **(C)** Milled acrylic disk used to fabricate the definitive music splint. **(D)** Frontal view of the completed definitive splint. **(E)** Lateral view of the completed definitive splint. **(F)** The definitive music splint in place.

A preliminary impression of the maxilla was obtained using irreversible hydrocolloid impression material (Hi-Technicol, GC, Tokyo, Japan). A frame was fabricated using a vacuum-adapted thermoforming technique with a 1.5 mm thick polyethylene terephthalate disk (Splint Clear 1.5 mm, Yamahachi Dental, Gamagori, Japan). Auto-polymerizing dental acrylic resin (Unifast III, GC, Tokyo, Japan) was added intraorally, and the device was adjusted according to the patient's perception of blowing comfort. The splint was then polished using an abrasive bur, producing a provisional music splint (Figure 2A).

After one month of use during musical activity, the provisional splint was reported to function without any issues. It was then digitized using a model scanner (D2000 Lab Scanner, 3Shape, Copenhagen, Denmark) to acquire three-dimensional data (Figure 2B). The definitive plumper-type music splint was designed using dental computer-aided design software (3Shape Dental System, 3Shape, Copenhagen, Denmark) and general-purpose 3D modeling software (Meshmixer, Autodesk, San Francisco, CA) based on the scanned data. The splint was subsequently fabricated via computer-controlled milling using a dental milling machine (DWX-52DC, DGSHAPE, Hamamatsu, Japan) and auto-polymerizing denture base acrylic resin (Palapress vario, Kulzer, Hanau, Germany) (Figure 2C) and polished (Figures 2D,E).

The definitive splint was delivered to the patient (Figure 2F) and adjusted according to his feedback while playing the instrument. Both subjective and objective evaluations were then performed.

### 2.3 Follow-up and outcomes

Performance evaluation included measurement of the maximum sustained note duration. The patient was instructed to sustain an E♭3 note at mezzo-forte (mf) and mezzo-piano (mp) levels, each repeated three times. The duration was measured in seconds, and the average value was recorded.

Subjective evaluation of playing comfort was conducted using a 10-point scale for the following items: mouthpiece adaptation, blowing efficiency, instrument stability, buccal stability, tone quality, loud note production, soft note production, high note production, low note production, short note production, long note production, and endurance. This scale was adapted from those used in previous studies, with additional items included based on the patient's feedback to better capture performance-related difficulties (4, 8, 9). Additional qualitative feedback regarding the overall playing experience was also obtained. The evaluation was repeated after one year of use during the patient's regular check-up.

**Figure 3:**
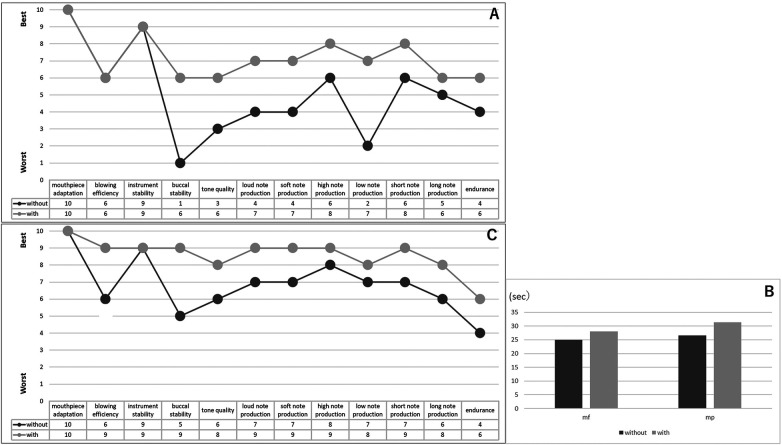
**(A)** The results of the questionnaire after using the definitive music splint for one month. “with” indicates performance with the music splint; “without” indicates performance without the music splint. **(B)** The maximum blowing time with and without using the music splint. **(C)** The results of the questionnaire after using the definitive music splint for one year.

The plumper-type music splint fit well, and the patient was able to use it in daily practice and on stage without any problems. He reported that he could practice for a longer time when the splint was worn, and no mucosal complications were observed on the soft tissues adjacent to the splint. Figure 3A shows the questionnaire results one month after delivery. Almost all items were rated favorably when the splint was worn, and even those that had been very low, such as buccal stability and low-tone production without the splint, showed marked improvement. Regarding qualitative feedback, the patient reported that without the splint his lips became fatigued and air leaked laterally, the sound sometimes became breathy or trembled, weak tones were difficult to produce as intended, and he became fatigued easily, which made him anxious. On the other hand, when the splint was worn, he felt that airflow was stabilized, pressure did not escape laterally, and he could articulate tones as intended, which gave him confidence. He also mentioned, however, that he was still struggling with the endurance and stability of his embouchure.

Figure 3B shows the results of the long-tone assessment, and Figure 3C shows the questionnaire results after one year of splint use. The maximum sound duration was longer with the splint in both mf and pp blowing. The questionnaire also indicated that, after one year of splint use, scores had improved even when the splint was not worn. In terms of qualitative feedback after adjustments, the patient reported that without the splint the tone lacked density and sometimes became breathy, whereas with the splint air leakage was prevented, intraoral pressure could be maintained, and he could concentrate on performance without unnecessary muscular effort. In addition, compared with the initial stage, he reported that he had become able to play with harder reeds, changing from 2½ to 3½, and he also felt that the facial surface remained more stable during blowing.

## 3 Discussion

In this case, the implant-supported prosthesis previously delivered at another clinic provided adequate function for mastication but presented difficulties during wind instrument performance. While oral functions such as speech, swallowing, and mastication are well recognized, the functional requirements for musical performance are often overlooked. Raising dentists’ awareness of other oral functions, such as singing and playing musical instruments, is desirable for future practice.

The use of a plumper-type music splint improved the patient's playing comfort compared with the non-use condition and reduced fatigue during performance, allowing the patient to participate confidently in orchestral activities. With the use of the plumper-type music splint, the patient's subjective evaluation improved to a level comparable to post-treatment conditions reported in previous case reports (4, 8, 9). Plumpers are usually used to compensate for intraoral space and to support soft tissues (11, 12). In this patient, difficulty in playing was attributed to the inability of the existing implant-supported full-arch prosthesis to fully restore the lost alveolar bone, even though the teeth were prosthetically reconstructed. Therefore, the use of a plumper-type device was well suited to address the functional issue in this case. No conclusions regarding reed hardness or facial symmetry can be drawn from a single case. Further investigation, including facial measurements during instrument performance (14), is warranted.

Several limitations should be acknowledged. Because retention of the plumper-type music splint relies on engaging the undercut by utilizing the flexibility of the material, its long-term durability with prolonged use remains uncertain. In addition, compared with the porcelain surface of the prosthetic superstructure, the material characteristics of the splint may predispose it to increased plaque accumulation, surface degradation, or discoloration over time. Nevertheless, as a removable device, a music splint poses fewer risks to performance compared with irreversible treatments, making it a suitable and low-risk option for wind instrument players (4). It is essential to design the splint according to the individual's oral conditions and to provide a device optimized for musical performance. It should also be noted that if a removable prosthesis had been selected instead of a fixed reconstruction, the prosthesis itself might have been more easily modified by adding or reducing material to accommodate musical performance (8). When planning prosthetic treatment for wind instrument players, treatment options should therefore be carefully discussed with the patient not only in terms of their general advantages and disadvantages, but also from the perspective of instrument performance.

## 4 Conclusion

In this case, the plumper-type music splint successfully compensated for the reduced intraoral tissue volume associated with the existing implant-supported prosthesis, resulting in improved embouchure stability and greater playing comfort. As a removable and minimally invasive approach, this appliance may serve as a practical adjunctive option for wind instrument players who develop performance-related functional difficulties after fixed full-arch implant rehabilitation.

## Data Availability

The original contributions presented in the study are included in the article/Supplementary Material, further inquiries can be directed to the corresponding author/s.
